# Age-specific symptom prevalence in women 35–64 years old: A population-based study

**DOI:** 10.1186/1471-2458-9-37

**Published:** 2009-01-26

**Authors:** Annika Bardel, Mari-Ann Wallander, Hans Wedel, Kurt Svärdsudd

**Affiliations:** 1 Uppsala University, Department of Public Health and Caring Sciences, Family Medicine and Clinical Epidemiology Section, Uppsala, Sweden; 2 Nordic School of Public Health, Gothenburg, Sweden

## Abstract

**Background:**

Symptom prevalence is generally believed to increase with age. The aim of this study was to evaluate the age specific prevalence of 30 general symptoms among Swedish middle-aged women.

**Methods:**

A cross-sectional postal questionnaire study in seven Swedish counties in a random sample of 4,200 women 35–64 years old, with 2,991 responders. Thirty general symptoms included in the Complaint Score subscale of the Gothenburg Quality of Life Instrument were used.

**Results:**

Four groups of age specific prevalence patterns were identified after adjustment for the influence of educational level, perceived health and mood, body mass index, smoking habits, use of hormone replacement therapy, and use of other symptom relieving therapy. Only five symptoms (insomnia, leg pain, joint pain, eye problems and impaired hearing) increased significantly with age. Eleven symptoms (general fatigue, headache, irritability, melancholy, backache, exhaustion, feels cold, cries easily, abdominal pain, dizziness, and nausea) decreased significantly with age. Two symptoms (sweating and impaired concentration) had a biphasic course with a significant increase followed by a significant decrease. The remaining twelve symptoms (difficulty in relaxing, restlessness, overweight, coughing, breathlessness, diarrhoea, chest pain, constipation, nervousness, poor appetite, weight loss, and difficulty in urinating) had stable prevalence with age.

**Conclusion:**

Symptoms did not necessarily increase with age instead symptoms related to stress-tension-depression decreased.

## Background

The burden of morbidity increases with increasing age, while the prevalence of symptoms seems to be less age dependent. If symptom prevalence mirrors disease prevalence then symptom prevalence should also increase with age. However, although symptoms may indicate disease they may also be attributable to the physiological effects of anxiety or other emotions arising from a personal problem [1]. Moreover, in addition to age, symptom reporting may be influenced by socioeconomic and lifestyle factors, such as educational level, physical activity, profession, income level, and social interaction [2-5].

Several studies have shown that women of all ages report more symptoms, take more of both prescription and non-prescription medication and consult physicians more often than men [6-8]. One possible explanation may be that women are more attentive to their wellbeing (physical and psychological), and therefore seek medical attention based on perception of their symptoms. Middle-aged women often present a variety of complaints at the onset of the menopause. It has been claimed that the menopausal syndrome is related more to personal characteristics than to the menopause per se, and that women in menopausal transition with several complaints of other origin than vasomotor may be suffering from underlying depression [9].

Complaint Score is a subscale of the Gothenburg Quality of Life instrument and contains a list of 30 symptoms. The subscale is intended to measure the tendency to report symptoms based on the assumption that symptom reporting reflects wellbeing. The instrument has been widely used during the last 30 years among men and women. It has been shown that symptom prevalence does not necessarily increase with age [1]. We decided to test the hypothesis that some symptoms increase with age whereas other have a decreasing or stable tendency in young to middle-aged women sampled from the general population.

## Methods

A random sample of 4200 women aged 35–64 years was drawn from the population register of the seven counties comprising the Uppsala-Örebro Health Care Region in central Sweden, a region large enough to be representative of Sweden. A postal questionnaire was sent to these women and 2991 (71.2%) responded. The age distribution of responders (49.6 ± 8.5 years) and non-responders (49.8 ± 8.7 years) was similar.

The questionnaire consisted of two parts. The first part contained information on height and weight, social background, lifestyle, medical history, gynaecological and obstetric history, and quality of life. Educational level was classified as compulsory education only, vocational training, high school, or college/university education. The women were classified according to their smoking habits as never-smokers, ex-smokers and current smokers. Body mass index was calculated as weight (kg)/height (m)2. The gynaecologic/obstetric data include number of pregnancies, ever use of contraceptives, menstrual status and ever undergone a hysterectomy or ooforectomy.

The Gothenburg Quality of Life Instrument was used to measure quality of life aspects [10]. For this report the Complaint Score and Well-being sub-scales were used. The Complaint Score is a list of 30 general symptoms. The women were asked to indicate which of the symptoms they had experienced during the past three months. Possible responses were "yes" or "no". At test-retest of the instrument a Spearman correlation coefficient of 0.54 was found even with seven years between test and retest and probably much higher with shorter interval. Of the well-being subscale questions, those on work situation, perceived health, physical fitness, mood, energy, patience, self-esteem, and being appreciated at home or outside the home were used in this study. The responses were given on seven-point interval scales ranging from "poor" (= 1) to "excellent, could not be better" (= 7). The corresponding test-retest correlation coefficient as above was 0.49 for mood and 0.46 for health.

The second part of the questionnaire focused on drugs prescribed during the past 12 months. For each prescription, information was collected on drug trade name, indication, dosage, duration of medication, and whether or not the woman was taking the drug currently. All prescribed drugs reported were coded according to the Anatomical Therapeutic Chemical Classification System (ATC) [11]. For this report, information on hormone replacement therapy (ATC codes G03C and G03F) and symptom relieving therapy (tranquilizers, hypnotics, antidepressants and painkillers with ATC codes N02A, N02B, N05B, N05C, N06A, M01A and M03B) was used. The study was approved by the Research Ethics Committee at Uppsala University.

### Statistical analysis

Statistical analyses were conducted using the SAS [12] programme package. First, preliminary analyses of symptom prevalence in relation to age were performed with univariate and multivariate logistic regression analyses, with symptom reporting (yes/no) as the dependent variable, and age and 18 other potential outcome affecting variables (education, physical fitness, smoking habits, body mass index, perceived work situation, health, mood, energy, self-esteem, patience, appreciated at home or outside the home, number of pregnancies, ever use of contraceptives, hormone replacement therapy, other symptom relieving medication, menstrual status, and hysterectomy or ooforectomy ever performed), as independent variables with backward elimination of non-significant variables. In addition, a square term for age and a number of potential interaction terms were tested.

A final set of independent variables (covariates) common for all symptoms and significantly related to most of them was then defined. In addition to age it included perceived health, perceived mood, educational level, body mass index, smoking habits, use of hormone replacement therapy, and use of other symptom relieving medication, and age squared for curvilinear functions. No interaction terms survived the preliminary analyses.

Based on the final logistic regression analyses, age specific symptom prevalence estimates, adjusted for the influence of the final set of covariates listed above, were computed. The fit of adjusted functions to crude data regarding functional form was excellent for all 30 symptoms. The adjustments only affected the prevalence levels and only moderately.

A power analysis was performed regarding the age effect on one symptom each in the prevalence increase, decrease and no change group. The power, given a study population of 2900, an alpha level of 0.01, and the slopes found in the study, was 99.9% for general fatigue and 90% for insomnia. For nervousness, where there was no significant change with age, more than 30,000 subjects would have been needed to reach 80% statistical power to detect an age related prevalence change.

All tests were two-tailed. In the preliminary analyses p < 0.05 was used. In the final analyses p < 0.01 for symptom trend across age was used to account for multiple testing.

## Results

Characteristics of the study population are given in Table 1. The mean age was 49.6 years, inter-quartile range 42–56. More than one fourth of the women had a university education and one fourth were smokers. Mean body mass index was 24.8, inter quartile ranged 22.2–26.8. The majority reported their mood and perceived health as moderately good or good. Hormone replacement therapy was currently used by 15%, and other symptomatic relieving medication by 13%.

**Table 1:** Characteristics.

	n	mean or %
Age (years)	2991	49.6 ± 8.5
Educational level		
Compulsory school only	831	28.5
Vocational school/high school	1299	44.5
College or university	787	27.0
Smoking habits		
Never smoked	1315	45.1
Ex-smoker	838	28.7
Current smoker	763	26.2
Body mass index (kg/(m^2^))		
15–24	1704	59.3
25–30	888	30.9
>30	201	9.8
Mood		
Poor (1–3)	207	7.1
Moderately good or good (4–6)	2182	74.6
Excellent (7)	534	18.3
Self-rated health		
Poor (1–3)	338	11.6
Moderately good or good (4–6)	2020	69.1
Excellent (7)	564	19.3
Hormone replacement therapy		
no use	2428	82.7
current use	441	15.0
past use	67	2.3
Symptom relieving therapy		
no use	2450	83.4
current use	375	12.8
past use	111	3.8

Psycho-socio-economic characteristics of the study population.

**Figure 1:**
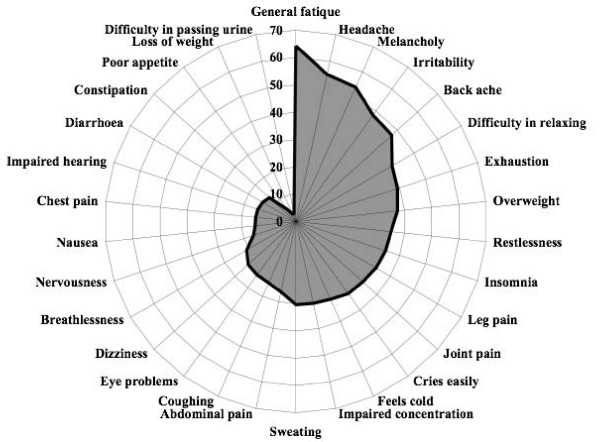
**Symptom prevalence**. Three months prevalence (%) of 30 symptoms among women 35–64 years of age.

**Figure 2:**
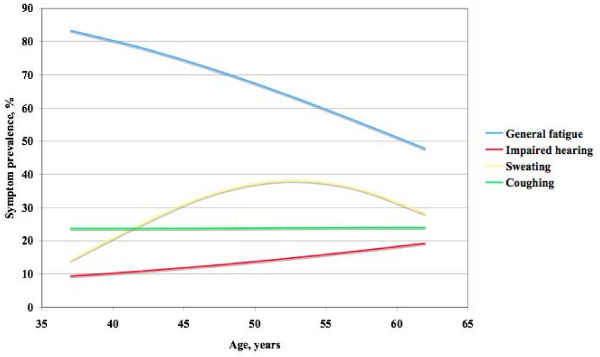
**Examples of prevalence course across age**. The four groups of symptom prevalence represented by four typical symptoms.

The prevalence of the 30 symptoms among all women in the study are shown in Figure 1. The most prevalent symptoms were general fatigue (64.2%), headache (54.9%), melancholy (53.7%), irritability (48.1%), and backache (47.1%). The least prevalent were difficulty urinating (3.1%), weight loss (3.1%), poor appetite (5.7%), constipation (13.0%), and diarrhoea (14.0%). The prevalence of the 30 symptoms by 5-year age groups, after adjustments for the influence of educational level, perceived health and mood, body mass index, smoking habits, use of hormone replacement therapy, and use of other symptom relieving therapy, is presented in Table 2. Four symptoms: insomnia, leg pain, eye problems and impaired hearing, all increased significantly by age with 8–10 percent from the youngest age group to the oldest age group. Joint pain has showed a similar but inconclusive increase. An example from this group, impaired hearing, is presented as a graph in Figure 2.

**Table 2:** Symptom prevalence data.

	Age groups						p across age *)
		
	35–39	40–44	45–49	50–54	55–59	60–64	
n	426	514	602	541	418	490	
Increasing prevalence							
Insomnia	28.1	30.0	32.0	34.1	36.2	38.4	<0.005
Leg pain	27.0	28.8	30.7	32.7	34.7	36.7	<0.005
Joint pain	27.0	28.5	30.1	31.7	33.3	35.0	
Eye problems	16.2	20.7	24.3	26.2	26.3	24.6	<0.005
Impaired hearing	9.4	10.9	12.6	14.6	16.8	19.3	<0.0001
Stable prevalence							
Difficulty relaxing	37.1	40.0	41.3	41.0	39.1	35.8	
Restlessness	35.3	34.4	33.5	32.5	31.6	30.7	
Overweight	32.5	32.8	33.2	33.5	33.8	34.1	
Coughing	23.7	23.7	23.8	23.9	24.0	24.0	
Breathlessness	16.6	17.1	17.5	18.0	18.5	18.9	
Diarrhoea	16.1	13.3	11.7	11.2	11.5	12.7	
Chest pain	12.2	12.3	12.5	12.6	12.8	13.0	
Constipation	12.2	12.1	11.9	11.8	11.6	11.5	
Nervousness	10.7	11.0	11.3	11.7	12.0	12.4	
Poor appetite	5.4	3.7	2.9	2.7	2.8	3.4	
Weight loss	2.6	2.5	2.3	2.2	2.0	1.9	
Difficulty urinating	2.2	2.2	2.2	2.2	2.2	2.2	
Biphasic prevalence							
Impaired concentration	28.1	31.3	32.2	30.7	27.0	21.6	<0.001
Sweating	14.1	25.0	34.1	38.1	35.9	28.2	<0.0001
Decreasing prevalence							
General fatigue	83.4	78.2	71.8	64.4	56.3	47.8	<0.0001
Headache	74.2	67.3	59.7	51.5	43.3	35.4	<0.0001
Irritability	66.1	59.1	51.8	44.4	37.2	30.5	<0.0001
Melancholy	60.1	58.3	56.4	54.6	52.7	50.8	<0.01
Backache	55.2	52.1	48.9	45.8	42.7	39.6	<0.0001
Exhaustion	45.4	45.6	43.2	38.1	30.9	22.5	<0.001
Feeling cold	40.7	36.0	31.6	27.5	23.7	20.3	<0.0001
Crying easily	38.6	35.5	32.4	29.5	26.8	24.3	<0.0001
Abdominal pain	32.2	28.7	25.4	22.3	19.6	17.1	<0.0001
Dizziness	27.3	24.9	22.6	20.6	18.6	16.8	<0.0005
Nausea	17.9	15.7	13.6	11.8	10.2	8.8	<0.0001

Symptom prevalence by age after adjustment for the influence of educational level, self-rated health and mood, body mass index, smoking habits, use of hormone replacement therapy, and use of other symptom relieving therapy.

*)p-values refer to prevalence trends across age.

Twelve symptoms, difficulty relaxing, restlessness, overweight, coughing, breathlessness, diarrhoea, chest pain, constipation, nervousness, poor appetite, weight loss, and difficulty urinating, had a stable prevalence with age, ranging from 41.3% to 1.9%. An example from this group, coughing, is also shown on the graph in Figure 2.

Two symptoms, impaired concentration and sweating, had biphasic prevalence. Impaired concentration increased from 28% among the youngest women, to a peak value of 32% at ages 45–49 and then decreased to 22% at age 60–64. Sweating had an even more pronounced biphasic course starting at 14% among the youngest, reaching a maximum level of 38% at ages 50–54 years and then decreasing to 28% among the oldest subjects. The prevalence course for sweating is shown in Figure 2.

The remaining eleven symptoms, general fatigue, headache, irritability, melancholy, backache, exhaustion, feeling cold, crying easily, abdominal pain, dizziness, and nausea, all showed a significantly decreasing prevalence with age. For many of the symptoms the prevalence at age 60–64 was half or less of what it was among the youngest women. General fatigue, shown in Figure 2, ranged from 83.4% among the youngest to 47.8% among the oldest women. Corresponding levels for headache were 74.2% and 35.5%, respectively, Table 2. Irritability ranged from 66.1% to 30.5% and backache from 55.2% to 39.6%.

## Discussion

The study revealed that the symptoms examined could be grouped into four prevalence patterns. Only five symptoms increased with age, twelve symptoms had stable prevalence, two had a biphasic course (first increasing and then decreasing) and eleven symptoms had decreasing prevalence with age after adjustment for the influence of the final set of covariates on symptom prevalence, thereby making prevalence estimates in various age groups more accurate and comparable.

This study was performed in a random population sample of women aged 35–64 years. The response rate was satisfactory (71%). However, the 29% non-responders may imply a potential selection bias in the estimation of the proportion of women reporting symptom, if responders and non-responder had different characteristics. The distribution of socioeconomic variables and drug use among the responders was similar to that of national samples in Sweden [13,14]. The size of the potential bias caused by non-response was estimated in a previous publication [15] and found to be of relatively little importance. Symptom reporting may have been affected by recall bias. However, the recall period of the last 3 months was short and the age range was such that younger and older women may be expected to have similar recall capacity.

The most commonly reported symptoms by the women in this study irrespective of age were general fatigue, headache, irritability, melancholy and backache, occurring in over or around 50% of the subjects. Furthermore, one third or more of the women had symptoms such as insomnia, restlessness, exhaustion and crying easily. Gorman has reviewed gender differences in the epidemiology and clinical presentations of depression [16]. In the United States, 1 out of 5 women suffered from an episode of major depression at some time in her life. Depression was about twice as common among women as among men. Women were more likely to have atypical symptoms of depression such as hypersomnia and hyperphagia, or to have co-morbid anxiety disorders or to attempt suicide than men.

It has previously been shown by Tibblin *et al.*, that there are different symptom prevalence patterns with age [1]. They studied 30 general symptoms in women and men and found generally higher symptom prevalence in women, using the same symptom questionnaire as used in this study. Three symptom patterns were found: increasing, decreasing, and biphasic prevalence, whereas four patterns, increasing, decreasing, biphasic, and stable were seen in the present study. The study by Tibblin *et al. *was performed some twenty years ago and no adjustments were made to make the age groups more comparable, which might explain the differences found in the two studies [1].

Five symptoms increased with age. It is a well-established fact [1] and is part of the normal ageing process, that hearing and vision deteriorate with age. Sleep disturbance has also been found by others to increase with age. It has been shown in a longitudinal study, that sleep duration decreased by 0.4 hours per night for women aged 38–66 years and that sleep problems increased by 30% for women aged 38–84 years [17,18].

In this study it was seen that leg pain as well as joint pain increased with age. This might partly be explained by increasing weight in those age groups. However, differences in body mass index between the groups were adjusted for, making overweight a less likely cause of these symptoms. Alternately, leg pain and joint pain could be signs of other musculoskeletal disorders, like osteoarthritis or rheumatoid arthritis, both of which have increasing prevalence with age [19,20].

One third of the symptoms studied had stable prevalence patterns. This is in contrast to prior findings where increasing, decreasing and biphasic courses have been reported [1]. We found stable prevalence patterns for difficulty in relaxing and nervousness, in contrast to the similar symptoms in the decreasing group. However, difficulty in relaxing and nervousness may indicate a personality trait rather than an age-related development. Also, symptoms like coughing, breathlessness and chest pain showed a similar unchanged pattern of relatively low prevalence, ranging from 12.2% to 23.7% among women ages 35 to 39 years indicating a more innocent nature of the symptoms than what it might have been in older age groups.

The prevalence of overweight, constipation, weight loss, and difficulty in urinating were also stable with age. Some of these symptoms, such as poor appetite, weight loss and difficulty in urinating, are commonly associated with serious diseases including diabetes and cancer, while constipation is common among the elderly. In this study overweigh ranged with age from 32.5% to 34.1%. This is broadly in line with what others found [21].

Two commonly considered groups of symptoms in women during the menopausal transition are psychological and vasomotor symptoms [22-27]. However, many symptoms believed to be related to the menopause also occur before and after this phase [15]. Only two symptoms in our study showed a biphasic pattern, possibly suggesting their association with the menopausal transition. Impaired concentration had a peak, with a maximum prevalence of 32.2% at the ages of 45–49 years, which is in agreement with findings reported by Tibblin *et al. *[1]. Increased prevalence of sweating was seen between the ages of 40 and 59, which is in line with what others have found [26,28].

Eleven symptoms had decreasing prevalence patterns with age: general fatigue, headache, irritability, melancholy, backache, exhaustion, feeling cold, crying easily, abdominal pain, dizziness and nausea. The prevalence for headache ranged from 74.2% among the youngest women to 35.4% among the oldest women in our study. It is known that headache prevalence decreases after 50 years of age [1], both for migraine and other types of headache [29].

A decrease in the prevalence of backache from 55.2% to 39.6% with age was seen in this study. Low back pain mostly occurs in an individual's most productive years, with a peak in the age interval 35–59 years [30-32], which is in line with the results presented. Abdominal pain and nausea decreased with age as well. These two symptoms were less common at the older end of the age range than at the younger end. In a recent UK primary care study the incidence of abdominal pain in women was seen to decrease between the ages of 35 and 60, where after which a slight increase could be seen [33].

The findings from the present study may be useful for public health administrators in forming health policies. They may also be used in a clinical setting where doctors are giving advice to their patients. Since the major part of the symptoms become less prevalent with age, other strategies than pharmacological treatment may come into perspective.

## Conclusion

Four prevalence courses across age were found. Few symptoms increased by age. The majority were either stable or decreased. Of those that decreased, the majority are generally associated with stress, strain or depression. The reasons why these symptoms generally decrease with age may be that women at the lower end of the age range have a larger burden with family, children and career than older women have, when the children may be grown up and their careers may be more stable. Symptoms do not necessarily increase with age, indicating that they may have other causes than ageing or disease.

## Competing interests

AB, HW, and KS none declared. MAW is an associate professor at Uppsala University and was at the time of the study employed by AstraZeneca R&D Mölndal (one of the funding agents).

## Authors' contributions

AB, MAW and KS designed the study. KS, AB and HW performed the analyses. All authors participated in the drafting and editing of the manuscript. All authors have seen and approved the final version.

## Pre-publication history

The pre-publication history for this paper can be accessed here:


